# Combined metabolic engineering of precursor and co-factor supply to increase α-santalene production by *Saccharomyces cerevisiae*

**DOI:** 10.1186/1475-2859-11-117

**Published:** 2012-08-31

**Authors:** Gionata Scalcinati, Siavash Partow, Verena Siewers, Michel Schalk, Laurent Daviet, Jens Nielsen

**Affiliations:** 1 Department of Chemical and Biological Engineering, Chalmers University of Technology, SE-412 96, Göteborg, Sweden; 2 Firmenich SA, Corporate R&D Division, CH-1211, Geneva 8, Switzerland

**Keywords:** Metabolic engineering, Isoprenoids, Sesquiterpenes, Continuous culture, *Saccharomyces cerevisiae*

## Abstract

**Background:**

Sesquiterpenes are a class of natural products with a diverse range of attractive industrial proprieties. Due to economic difficulties of sesquiterpene production via extraction from plants or chemical synthesis there is interest in developing alternative and cost efficient bioprocesses. The hydrocarbon α-santalene is a precursor of sesquiterpenes with relevant commercial applications. Here, we construct an efficient *Saccharomyces cerevisiae* cell factory for α-santalene production.

**Results:**

A multistep metabolic engineering strategy targeted to increase precursor and cofactor supply was employed to manipulate the yeast metabolic network in order to redirect carbon toward the desired product. To do so, genetic modifications were introduced acting to optimize the farnesyl diphosphate branch point, modulate the mevalonate pathway, modify the ammonium assimilation pathway and enhance the activity of a transcriptional activator. The approach employed resulted in an overall α-santalene yield of a 0.0052 Cmmol (Cmmol glucose)^-1^ corresponding to a 4-fold improvement over the reference strain. This strategy, combined with a specifically developed continuous fermentation process, led to a final α-santalene productivity of 0.036 Cmmol (g biomass)^-1^ h^-1^.

**Conclusions:**

The results reported in this work illustrate how the combination of a metabolic engineering strategy with fermentation technology optimization can be used to obtain significant amounts of the high-value sesquiterpene α-santalene. This represents a starting point toward the construction of a yeast “sesquiterpene factory” and for the development of an economically viable bio-based process that has the potential to replace the current production methods.

## Background

**Figure 1:**
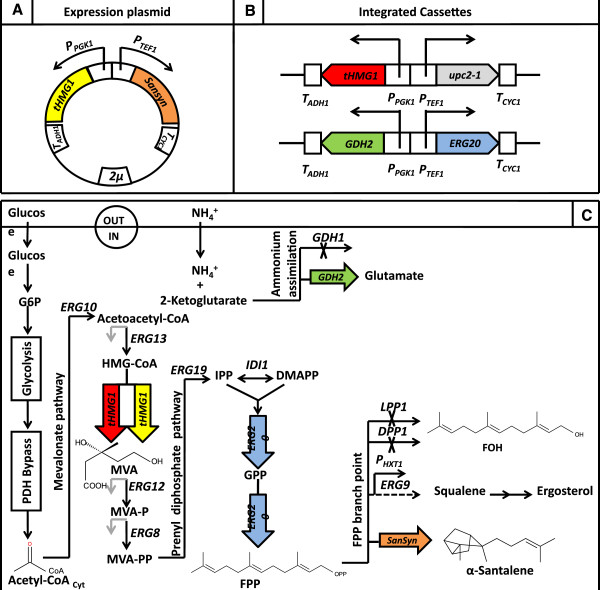
**Genetic engineering approach for increasing α-santalene production.** (**A**) Expression plasmid pISP15 containing *tHMG1* encoding truncated HMG-CoA reductase, a codon optimized santalene synthase gene (*SanSyn*_*opt*_) P_*TEF1*_ and P_*PGK1*_ promoters as well as T_*ADH1*_ and T_*CYC1*_ terminator sequences. (**B**) Integrated cassettes, rectangles containing arrows represent the promoters and their directionality, pentagons the genes and empty squares the terminators. (**C**) Scheme of the engineered mevalonate, prenyl phosphate and ammonium assimilation pathways and FPP branch point; overexpressed and deleted genes are highlighted. Pathway intermediates: G6P: glucose-6-phosphate, Acetyl-CoA_cyt_: cytosolic acetyl-CoA, HMG-CoA: 3-hydroxy-3-methylglutaryl-CoA, MVA: mevalonate, MVA-P: phosphomevalonate, MVA-PP: diphosphomevalonate, IPP: isopentenyl diphosphate, DMAPP: dimethylallyl diphosphate, GPP: geranyl diphosphate, FPP: farnesyl diphosphate, FOH: farnesol. Overexpressed genes are *tHMG1* (encoding truncated HMG-CoA reductase), *ERG20* (encoding FPP synthase), *GDH2* (encoding NAD-dependent glutamate dehydrogenase), and *SanSyn*_*opt*_ (encoding α-santalene synthase). Deleted genes are *GDH1* (encoding NADP-dependent glutamate dehydrogenase), *LPP1* and *DPP1* (both encoding lipid phosphate phosphatases). The promoter of the *ERG9* gene (encoding squalene synthase) is replaced with P_*HXT1*_. Genes whose promoters contain Upc2 binding sites are indicated with a grey arrow: *ERG13* (encoding HMG-CoA synthase), *ERG12* (encoding mevalonate kinase), and *ERG8* (encoding phosphomevalonate kinase). Additional genes indicated are *ERG10* (encoding acetoacetyl-CoA thiolase), *ERG19* (encoding diphosphomevalonate decarboxylase) and *IDI* (encoding IPP isomerase).

Isoprenoids are a class of natural compounds with many potential commercial applications (e.g. flavoring agents, fragrances, food colorants, pharmaceutical agents and biofuel precursors), and there has recently been much interest in biotechnological production of these compounds [1-4]. Limitations in raw material accessibility, low yields and high costs of the current isoprenoid production through plant extraction or difficulties with chemical synthesis have caused interest in engineering cell factories that can be used to produce isoprenoids in cost competitive bioprocesses [5-7]. Isoprenoids are natively produced in yeast though the mevalonate (MVA) pathway in which the universal isoprene functional unit isopentenyl diphosphate (IPP) is produced from acetyl-CoA (Figure 1) [8]. The terminal product IPP and its isomer dimethylallyl diphosphate (DMAPP) are subsequently condensed in the prenyl diphosphate pathway generating isoprene derivatives of different chain length (C_5_-C_20_) [9]. The sesquiterpene hydrocarbon α-santalene is a precursor of commercially relevant sesquiterpenes (C_15_) and it is generated in a one-step conversion from the intermediate building block farnesyl diphosphate (FPP) [10]. Stoichiometry of α-santalene (C_15_H_24_) production in *S. cerevisiae* via the MVA pathway in purely oxidative growth conditions can be summarized as:

$$\;\begin{array}{c} -4.{5\;\text{C}}_{6}{\text{H}}_{12}{\text{O}}_{6}-\text{9 ATP}–\text{6 NADPH}+{\text{C}}_{15}{\text{H}}_{24}+\\ \;\text{18 NADH}+{\text{12 CO}}_{2}=0_{\;} \end{array}$$

which demonstrates that α-santalene production involves a net consumption of ATP and NADPH, whereas there is a net production of NADH.

Considerable efforts have been made to engineer yeast for isoprenoid production [8,11]. Recently, progress has been reported in developing a *S. cerevisiae* strain capable to produce commercially relevant amounts of α-santalene [12], and the aim of the present work was to develop a *S. cerevisiae* production platform for sesquiterpene compounds that could serve as an inexpensive, environmentally compatible alternative to current production methods. We undertook a multistep metabolic engineering strategy combining four different approaches to increase α-santalene production. These included: (i) Modulation and optimization of the FPP branch point (ii) Modulation of the MVA pathway to increase the precursor pool for isoprenoid synthesis (iii) Increasing the availability of the reductive cofactor NADPH by modifying the ammonium assimilation pathway and (iv) Enhancing the activity of a transcriptional activator of sterol biosynthesis.

(i) In order to minimize the overflow to the biosynthetically related sterols that have the same precursor as α-santalene, FPP, the native promoter (P_*ERG9*_) of squalene synthase (SQS) was replaced with a glucose sensing P_*HXT1*_ promoter [12]. Previous attempts to increase cytosolic FPP availability by down-regulating the *ERG9* gene resulted in a rapid dephosphorylation of FPP to farnesol (FOH) [13-15]. To minimize the flux towards farnesol two genes, *LPP1* and *DPP1,* encoding enzymes with FPP dephosphorylation activity have been deleted [12,16], and we also adapted this approach here (Figure 1).

(ii) As a second part of the strategy we amplified the flux through the MVA pathway by engineering two key enzymatic steps. The mevalonate producing 3-hydroxy-3-methyl-glutaryl-CoA reductase (HMGR) enzyme is a highly regulated enzyme and is generally believed to exert a high degree of flux control in the MVA pathway. Part of its regulation is via the N-terminal domain of Hmg1 that spans the membrane of the endoplasmic reticulum (ER) and hereby interacts with sterol sensing components of the ER membrane. This feed-back regulation by sterols can be eliminated by expressing a modified form of HMGR lacking the trans-membrane region [17]. Here we used a genetic modification widely used in the past in order to circumvent post-transcriptional regulation of HMGR [18]. The *HMG1* gene region coding for the catalytic domain was over-expressed resulting in a constitutively active, cytosolic variant of Hmg1. This strategy has been successfully used before for over-producing several different isoprenoids in *S. cerevisiae*[12,14,19-21]. The other enzymatic step engineered in the MVA pathway was the one mediated by farnesyl diphosphate synthase (FPPS) (encoded by the essential gene *ERG20*), which catalyses the condensation of IPP units into geranyl diphosphate (GPP) and FPP [22]. IPP condensing enzymes are interspecies conserved and the yeast *ERG20* gene product evolved towards specific production of FPP rather than GPP [23]. Due to the pivotal nature of the FPP molecule as precursor of many essential compounds such as dolichol, ubiquinone, isoprenylated proteins and ergosterol [24] its synthesis by FPPS is tightly regulated and has been identified as a flux controlling step of the MVA pathway, in particular controlling the intracellular FPP availability and its distribution into derived products [25,26]. The efficiency of *ERG20* overexpression to increase the level of IPP conversion to FPP and its derivatives depends, however, on the growth conditions employed and the yeast background strain utilized [20,27]. In this study, the effect of overexpressing *ERG20* on α-santalene production has been investigated.

(iii) The manipulation of the NADH and NADPH cofactor balance in order to overcome limits imposed from the cellular redox constraints is a well-established metabolic engineering strategy [28]. The reaction leading to α-santalene formation results in net production of NADH and consumption of NADPH (see reaction above). A change in the NADH:NADPH ratio in favor of NADPH would therefore be beneficial for product formation. Increasing the availability of reduced cofactor NADPH by deleting the NADPH consuming reaction of glutamate dehydrogenase encoded by *GDH1* has previously been applied to improve product formation [29]. Similarly, activation of an alternative ammonium utilization route in a *gdh1*Δ strain by overexpressing the NAD-dependent glutamate dehydrogenase encoded by *GDH2* resulted in an increase of NADH consumption during the anabolic process and in a modification of the yeast cofactor balance [30]. More recently, *in silico* analysis identified the same strategy as an approach to increase sesquiterpene production [31]. Here we evaluated the effect of *GDH1* deletion alone as well as coupled with simultaneous over-expression of *GDH2* on α-santalene production (Figure 1).

(iv). The last strategy we employed involved engineering of a key transcription factor with the objective to generally up-regulate expression of the MVA pathway genes. Upc2 and Ecm22 have been identified as the main transcription factors responsible for the activation of several MVA and ergosterol pathways genes [32]. The point mutation *upc2-1* discovered first for conferring the ability to assimilate extracellular sterols during aerobic cultivation [33] has been demonstrated to result in a constitutively active form of Upc2 [34]. Overexpression of *upc2-1* has been employed to transcriptionally up-regulate the MVA pathway genes during isoprenoid production, but its effect on enhancing the carbon flow through the pathway was modest when used alone [19,20]. However, when combined together with *ERG9* down-regulation, it produced a clear increase in total isoprenoid production [20,35]. In the current work, contribution of the *upc2-1* overexpression on the production of α-santalene was tested in combination with the modifications described above.

**Figure 2:**
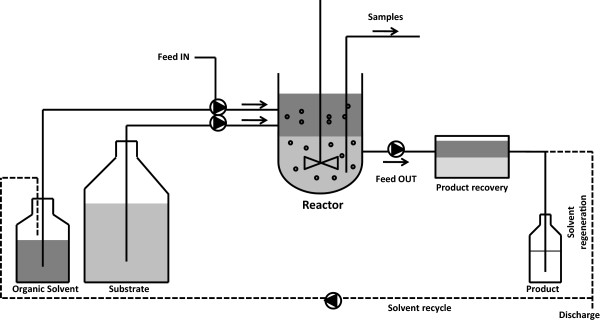
**Set-up of the *in situ *product removal (ISPR) chemostat cultivation process.** A stirred tank reactor is operated in continuous cultivation mode as double phase system feeding culture medium (light grey) and organic solvent (dark grey). The product is continuously captured in the organic phase due to its high hydrophobicity. In an integrated downstream step the two phases of the effluent are partitioned in a settler. Subsequently, the product is recovered from the organic phase, which can then be further recycled in the same process. The exhausted medium is discarded.

Although these strategies have been employed before for increasing sesquiterpene production in yeast, they are here for the first time combined in a single strain. All the genetic modifications described above were integrated into the yeast genome to enhance the genetic stability of the production strain during long term cultivation. However, in order to ensure flexibility and to allow the platform strain to be used for production of a range of different isoprenoids, we expressed the synthase gene required for the final conversion of FPP into α-santalene together with an additional copy of *tHMG1* on a multicopy plasmid (Figure 1). The effect of the different metabolic engineering strategies on isoprenoid production was evaluated using an integrated fermentation/downstream recovery process with a two-phase partitioning continuous cultivation set-up (Figure 2). By combining the different strategies we developed a yeast strain and a fermentation process that resulted in high sesquiterpene titers and the results represent a first step toward the long term goal of establishing an efficient sesquiterpene production process.

## Results

The primary objective of this study was to enhance the availability of intracellular FPP to increase the production level of the sesquiterpene α-santalene and to evaluate the metabolic response of *S. cerevisiae* to the genetic modifications. A double phase continuous cultivation method was developed as production process to investigate the performances of the engineered strains at glucose-limited conditions.

### Characterization of engineered sesquiterpene producing strains in two-phase chemostat cultivation

**Figure 3:**
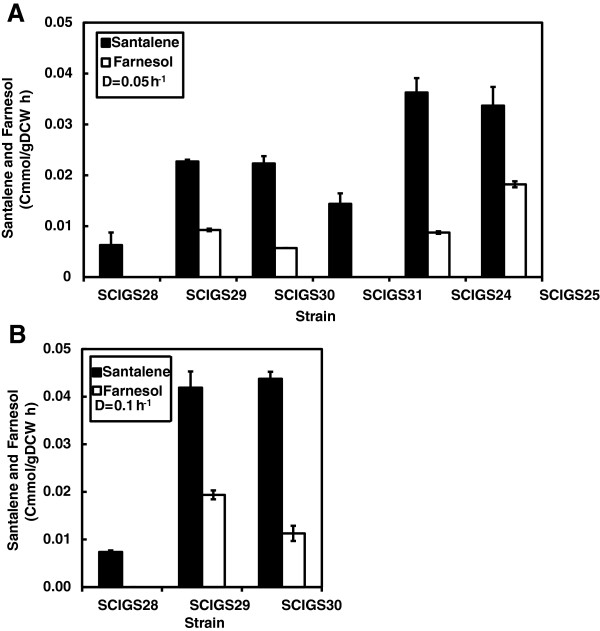
**Sesquiterpene productivity in a two-phase partitioned glucose-limited aerobic chemostat.** α-Santalene and farnesol production rate in Cmmol (g biomass)^-1^ h^-1^ (the C-molar weight of α-santalene and farnesol are, respectively, 13.62 and 14.82 g Cmol^-1^). (**A**) Strains SCIGS28 (*tHMG1*↑), SCIGS29 (+ P_*HXT1*_*-ERG9, lpp1*Δ), SCIGS30 (+ *dpp1*Δ), SCIGS31 (+ *gdh1*Δ), SCIGS24 (+ *ERG20*↑*, GDH2*↑), SCIGS25 (+ *upc2-1*↑*, tHMG1*↑) cultivated at dilution rate D = 0.05 h^-1^. (**B**) Strains SCIGS28 (*tHMG1*↑), SCIGS29 (+ P_*HXT1*_*-ERG9; lpp1*Δ), SCIGS30 (+*lpp1*Δ) cultivated at dilution rate D = 0.1 h^-1^. Error bars represent the standard deviation from three independent cultivations.

**Figure 4:**
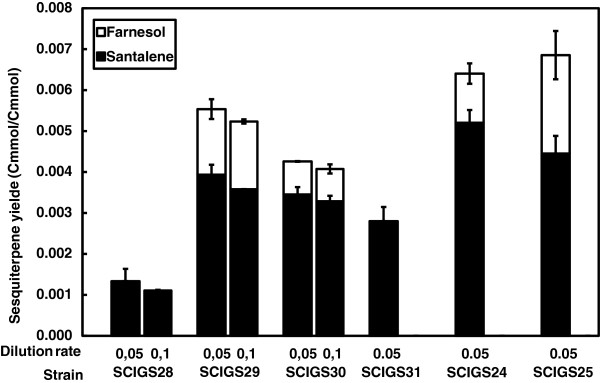
**Sesquiterpene yield in a two-phase partitioned glucose limited aerobic chemostat.** α-Santalene and farnesol yield in Cmmol (Cmmol glucose)^-1^. Strains SCIGS28 (*tHMG1*↑), SCIGS29 (+ P_*HXT1*_*-ERG9, lpp1*Δ), SCIGS30 (+ *dpp1*Δ) were cultivated at dilution rate D = 0.05 h^-1^ and D = 0.1 h^-1^. Strains SCIGS31 (+ *gdh1*Δ), SCIGS24 (+*ERG20*↑*, GDH2*↑), SCIGS25 (+ *upc2-1*↑*, tHMG1*↑) were cultivated at dilution rate D = 0.05 h^-1^. Error bars represent the standard deviation from three independent cultivations.

**Figure 5:**
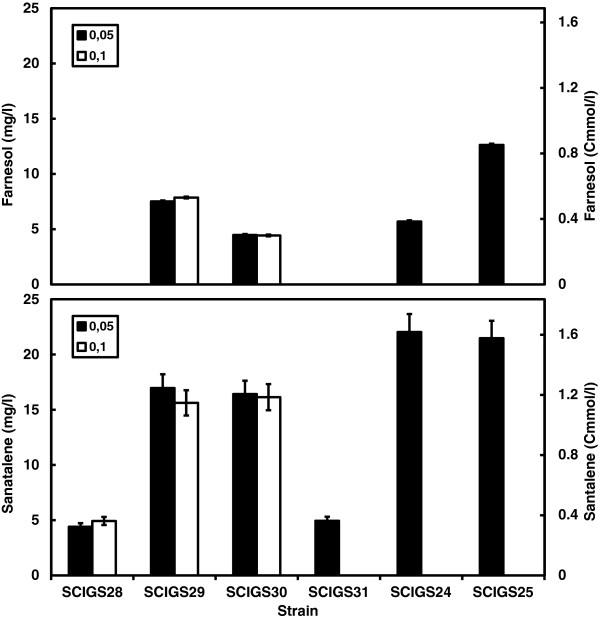
**α-Santalene titers in a two-phase partitioned glucose limited aerobic chemostat.** α-Santalene (bottom) and farnesol (top) titers expressed in mg l^-1^ and Cmmol l^-1^ (the C-molar weight of α-santalene and farnesol are respectively 13.62 and 14.82 g Cmol^-1^). Strains SCIGS28 (*tHMG1*↑), SCIGS29 (+ P_*HXT1*_*-ERG9, lpp1*Δ), SCIGS30 (+ *dpp1*Δ) were cultivated at dilution rate D = 0.05 h^-1^ and D = 0.1 h^-1^. Strains SCIGS31 (+ *gdh1*Δ), SCIGS24 (+*ERG20*↑*, GDH2*↑), SCIGS25 (+ *upc2-1*↑*, tHMG1*↑) were cultivated at dilution rate D = 0.05 h^-1^. Error bars represent the standard deviation from three independent cultivations.

*S. cerevisiae* was engineered to produce α-santalene by introducing the expression plasmid pISP15 containing a copy of *tHMG1* and codon optimized *SanSyn* (*SanSyn*_*opt*_) under control of the *PGK1* and *TEF1* promoters, respectively (strain SCIGS28). The transformed strain was initially tested for its α-santalene producing capacity in a double-phase chemostat process at a dilution rate of 0.05 h^-1^ resulting in an α-santalene yield of 0.0013 Cmmol (Cmmol glucose)^-1^ and a production rate of 0.006 Cmmol (g biomass)^-1^ h^-1^ (corresponding to 0.086 mg (g biomass)^-1^ h^-1^). All the following strain development strategies were assessed based on the yield and productivity of this control strain (SCIGS28) and are reported in Figures 3 and 4, whereas the titers are given in Figure 5.

Replacement of the native P_*ERG9*_ promoter with *P*_*HXT1*_ was previously proven to efficiently reduce ergosterol production and increase the availability of FPP for the conversion into sesquiterpene products [12]. Here, the same modification was introduced in an *lpp1*Δ strain carrying the expression vector resulting in strain SCIGS29. Using P_*HXT1*_ to control *ERG9* expression combined with *LPP1* deletion resulted in an increase in α-santalene yield and productivity of 3- and 3.8-fold, respectively. α-Santalene production was accompanied by the formation of the FPP-derived farnesol (FOH) at a production rate of 0.006 Cmmol (g biomass)^-1^ h^-1^ (Figure 3A). The impact of the additional deletion of *DPP1* was tested in an attempt to reduce the rate of hydrolysis of FPP into the undesired by-product FOH (strain SCIGS30). This resulted in an almost unchanged flux towards α-santalene formation, but in a reduction of the farnesol yield and productivity by 50% and 44%, respectively.

In a following approach, the impact of perturbing the redox metabolism on α-santalene accumulation was evaluated introducing the deletion of *GDH1* encoding NADP-dependent glutamate dehydrogenase (strain SCIGS31). In the strain harboring the additional *GDH1* deletion, no further enhancement in α-santalene productivity was detected. Interestingly, no substantial FOH formation was detected in this strain (Figure 3A and 4).

Subsequently, we monitored the effect of activating an NADH consuming reaction for ammonium assimilation together with the up-regulation of the second MVA pathway flux controlling step FPPS, integrating and over-expressing simultaneously the genes *GDH2* and *ERG20* (strain SCIGS24). This combination resulted in a significant increase of sesquiterpene production contributing to attain the maximum α-santalene yield and productivity of 0.0052 Cmmol (Cmmol glucose)^-1^ and 0.036 Cmmol (g biomass)^-1^ h^-1^, respectively. The additional up-regulation of *GDH2* and *ERG20* combined with all previous features produced a 4- and 6-fold improvement, respectively, in α-santalene yield and productivity compared to the control strain (Figure 3A and 4).

The MVA pathway was further engineered by integrating into the yeast genome the mutated transcription factor gene *upc2-1* and an extra copy of *tHMG1* (strain SCIGS25). Previously, both strategies, using an additional genome integrated copy of *tHMG1* next to plasmid-based expression and the over-expression of *upc2-1* have displayed little or only a strain-dependent effect on final product production [14,20]. Similarly, our combined approach did not contribute to increase α-santalene production over the best producing strain obtained, SCIGS24. However, in contrast to the insignificant change in α-santalene productivity strain SCIGS25 exhibited a 2-fold increase in FOH formation yielding a final FOH yield of 0.0024 Cmmol (Cmmol glucose)^-1^ and a productivity of 0.018 Cmmol (g biomass)^-1^ h^-1^. It is therefore worth mentioning that strain SCIGS25 reached the highest total sesquiterpene yield and productivity of 0.0069 Cmmol (Cmmol glucose)^-1^ and 0.052 Cmmol (g biomass)^-1^ h^-1^ (santalene + farnesol), respectively (Figure 3A and 4).

### Evaluation of sesquiterpene production strains at different dilution rates

Under the employed conditions, the engineered strains exhibited significant changes in the total amount of sesquiterpene produced. The sesquiterpene productivity level varied almost 10-fold between the strains, from 0.006 to 0.052 Cmmol (g biomass)^-1^ h^-1^. Chemostat cultivation mode offers the advantage of manipulating with accuracy the dilution rate, which at these conditions is equal to the specific growth rate [36]. We therefore decided to investigate the behavior of the sesquiterpene production strains at two different growth rates. All previous cultivations were performed at a dilution rate of 0.05 h^-1^ and when the control strain was grown at D = 0.1 h^-1^, a small decrease in the α-santalene yield was observed (Figure 4) whereas its productivity remained essentially unchanged (Figure 3). The increase in α-santalene production observed for strains SCIGS29 and SCIGS30 at low dilution rate (D = 0.05 h^-1^) was also seen at the higher dilution rate of 0.1 h^-1^. α-Santalene productivities measured for these strains were, respectively, 0.041 and 0.043 Cmmol (g biomass)^-1^ h^-1^ representing a 6-fold increase compared to the control strain and almost a 2-fold increase compared to the productivity at D = 0.05 h^-1^ (Figure 3B). In contrast, the yield was slightly reduced. Consistently, the *DPP1* deletion resulted in reduced FOH accumulation in strain SCIGS30 compared to the *lpp1*Δ single deletion (strain SCIGS29). The ratios between the α-santalene and the farnesol yield in the two strains of 2.3 and 4.2, respectively, were maintained when the dilution rate was raised to 0.1 h^-1^ (Figure 4). Consistently, the same product proportion was also seen in the productivities (Figure 3). Therefore, the distribution of FPP between the two products remained unchanged when the dilution rate was increased.

Surprisingly, strains SCIGS31, SCIGS24 and SCIGS25 were unable to sustain growth at D = 0.1 h^-1^ and cultures were washed out (see section below).

### Strain physiology in batch and chemostat cultivation

In order to evaluate if the modifications applied to increase sesquiterpene production affected yeast physiology a detailed characterization of the recombinant strains was carried out. Control strain SCIGS28 displayed a fully respiratory metabolism (RQ = 1.0) under both dilution rates. The principal physiological parameters (e.g. Y_sx_, r_s_, r_CO2_ and r_O2_) were comparable with the wild type strain CEN.PK113-7D [37,38]. Strains SCIGS29 and SCIGS30 exhibited major alterations in their physiology. An increase in the residual glucose concentration of 6.4 fold at D = 0.05 h^-1^ and 2.5 fold at D = 0.1 h^-1^ was observed for both strains. As direct consequence of the increase in the residual glucose concentration aerobic fermentation set in, resulting in ethanol formation accompanied with acetate accumulation. A marked reduction in the biomass yield from 0.5 to 0.29-0.28 (D = 0.05 h^-1^) and 0.28-0.25 g biomass (g glucose)^-1^ (D = 0.1 h^-1^) was measured for the two strains (Table 1). However, only a small fraction corresponding to 4% (Cmmol products (Cmmol glucose)^-1^) of the glucose consumed was fermented to ethanol and acetate. Additionally, a clear increase in the glucose (r_s_) and oxygen consumption rate (r_O2_) and carbon dioxide production rate (r_CO2_) was observed (Table 1). This physiological response was observed at both D = 0.05 and 0.1 h^-1^. Despite several attempts, it was not possible to achieve a steady-state when strains SCIGS31, SCIGS24 and SCIGS25 were grown at D = 0.1 h^-1^. Instead, a progressive decrease of the biomass concentration over time was observed consistent with wash-out kinetics. The following characterization for these strains was therefore conducted only at D = 0.05 h^-1^.

**Table 1:** Physiological parameters measured during double-phase chemostat cultures of strains SCIGS28, SCIGS29, SCIGS30, SCIGS31, SCIGS24 and SCIGS25

**Strain**	***D***	***Y***_***xs***_	**r**_**s**_	**r**_**CO2**_	**r**_**O2**_	**r**_**etoh**_	**r**_**acet**_	**RQ**	**C**_**s**_	**C**_**balance**_	**Tot**_**sant**_
	**(h**^**-1**^**)**	**(g g**^**-1**^**)**	**(mmol g biomass**^**-1**^**h**^**-1**^**)**					**(r**_**CO2**_**/r**_**O2**_**)**	**(mM)**	**(%)**	**(mg 24 h**^**-1**^**l**^**-1**^**)**
SCIGS28	0.051 ±0.002	0.50 ±0.01	0.57 ±0.01	1.12 ±0.08	1.12 ±0.06	0	0	1.00 ±0.02	0.18 ±0.02	100.3 ±2.1	11.8 ±0.3
	0.10 ±0.01	0.50 ±0.01	1.11 ±0.03	2.67 ±0.15	2.49 ±0.07	0	0	1.07 ±0.05	0.16 ±0.01	101.9 ±1.1	5.2 ±0.2
SCIGS29	0.050 ±0.003	0.29 ±0.01	0.97 ± 0.04	3.17 ±0.04	2.95 ±0.15	0.082 ±0.001	0.024 ±0.001	1.07 ±0.07	1.16 ±0.04	96.9 ±3.2	37.6 ±0.2
	0.10 ±0.01	0.28 ±0.01	1.95 ±0.05	6.40 ±0.18	5.51 ±0.20	0.128 ±0.004	0.039 ±0.008	1.16 ±0.22	0.39 ±0.01	95.0 ±0.9	20.4 ±0.1
SCIGS30	0.051 ±0.001	0.28 ±0.02	1.09 ±0.05	3.60 ±0.09	3.41 ±0.22	0.099 ±0.015	0.024 ±0.003	1.05 ±0.09	1.15 ±0.04	94.7 ±0.4	39.4 ±0.4
	0.10 ±0.02	0.25 ±0.01	2.26 ±0.09	7.70 ±0.19	6.37 ±0.29	0.161 ±0.007	0.032 ±0.005	1.21 ±0.14	0.40 ±0.01	93.5 ±1.2	20.1 ±0.1
SCIGS31	0.051 ±0.001	0.33 ±0.01	0.86 ±0.01	2.31 ±0.08	1.42 ±0.23	0.501 ±0.077	0.047 ±0.007	1.62 ±0.11	30.67 ±0.78	105.4 ±4.9	6.1 ±0.1
SCIGS24	0.051 ±0.001	0.24 ±0.01	1.16 ± 0.03	3.85 ±0.05	3.21 ±0.07	0.185 ±0.003	0.020 ±0.008	1.20 ±0.02	2.53 ±0.09	93.7 ±5.3	26.9 ±0.2
SCIGS25	0.048 ±0.003	0.21 ±0.01	1.26 ±0.02	4.41 ±0.04	3.82 ±0.04	0.195 ±0.005	0.027 ±0.007	1.16 ±0.03	2.91 ±0.14	93.8 ±2.9	24.9 ±0.3

*μ*_*max*_, specific growth rate (h^-1^); *D*, dilution rate (h^-1^); *Ysx*, biomass yield (g biomass (g substrate)^-1^); specific consumption rates of glucose(r_s_), and oxygen (r_O2_) (mmol (g biomass)^-1^ h^-1^); specific production rates of carbon dioxide (r_CO2_), ethanol (r_etoh_),and acetate (r_acet_) (mmol (g biomass)^-1^ h-^1^). RQ, respiratory quotient r_CO2_/r_O2_; C_s_, residual glucose concentration (mM); C_balance_, carbon recovery (%); Tot_sant_ total amount of α-santalene produced (mg 24 h^-1^ l^-1^). Values represent the mean ± S.D. of three independent cultivations.

When deletion of *GDH1* was introduced (strain SCIGS31) a considerable fraction of the glucose, 31 mmol l^-1^, was recovered corresponding to a consumption of only 33% of the total sugar provided. However, it was still possible to reach a steady state. In this strain, the rate of alcoholic fermentation increased to 0.51 mmol (g biomass)^-1^ h^-1^ and the metabolism shifted more predominantly to a respiro-fermentative state (RQ = 1.62), where 21% of the carbon source was metabolized to the fermentation products ethanol and acetate. These pronounced metabolic changes were probably related to a limitation in ammonium consumption as a consequence of Gdh1 inactivation.

Overexpression of *GDH2* is known to partially complement the ammonium assimilation defect in a *gdh1*Δ strain [30] and resulted in a clear reduction of the ethanol and acetate production rate in strains SCIGS24 and SCIGS25 compared to strain SCIGS31. It is worth noticing that in strain SCIGS24, a large decrease in the biomass yield occurred and the specific glucose and O_2_ consumption rates and the CO_2_ production rate increased respectively to a value of 1.16, 3.21 and 3.85 mmol (g biomass)^-1^ h^-1^. The previously described overflow metabolism phenomenon towards fermentation products was also observed in strains SCIGS24 and SCIGS25 and led to a fraction of carbon fermented to ethanol and acetate close to 6% for both strains. All engineered strains except the control strain exhibited overflow metabolism under the tested conditions. The fraction of glucose converted into fermentation products ranged between 0.04 and 0.21 Cmmol products (Cmmol glucose)^-1^. If strain SCIGS31 (which exhibited a behavior different from all other strains probably related to the major role played by the ammonium limitation) is excluded from this consideration, it is interesting to notice that the ratios of the different fermentation products measured vary substantially between the strains. A significantly higher ethanol: acetate ratio was observed for strains SCIGS24 and SCIGS25 compared to SCIGS29 and SCIGS30 indicating a redistribution of flux around the pyruvate dehydrogenase (PDH) bypass at the acetaldehyde level. The increased ethanol: acetate ratio was reflected in an increase in the formation of sesquiterpene products, which are derived directly from the cytosolic acetyl-CoA produced through the pyruvate decarboxylase route. On the other hand, the engineered strains showed a clear decrease in biomass yield compared to the control strain suggesting a carbon flux redirection towards other products. The fraction of carbon lost in the drop of biomass yield could not be accounted for in the residual unconsumed glucose or in the fermentation products. Instead, carbon dioxide was the main carbon product. Surprisingly, the increase in ethanol and acetate productivity was not related to any decrease in the respiration rate. The oxygen uptake rate was increased in all engineered strains compared to the control strain and reached the highest value of 4.41 mmol (g biomass)^-1^ h^-1^ in strain SCIGS25 suggesting a strong reprogramming of cell metabolism in these strains.

## Discussion

In this study, we provide an example of several rounds of metabolic engineering aimed at increasing the production of the commercially relevant sesquiterpene compound α-santalene. The strain improvement strategy was combined with development of a cost effective fermentation process based on a two-phase continuous cultivation mode.

### Double-phase chemostat as a tool to study metabolically engineered strains

Continuous cultivation modes have been employed in industrial bioprocesses (e.g. insulin production) and offer several advantages compared to batch conditions [36]. One is that they allow a precise comparison of productivities of selected genetically engineered strains under well-controlled constant conditions and to explore the effect of the growth rate independently of the other parameters.

Being extensively used in bioprocesses to produce aroma compounds, *in situ* product removal (ISPR) (for review see [39]) was applied in this study to maximize the product recovery. Through the combination of ISPR with chemostat cultivations we obtained a production system that offers the advantage of continuous recovery of the product in the fermented effluent from the selected organic phase which can subsequently be recycled, regenerated and reused in the same process for a prolonged time of cultivation (for review see [40]) The developed set-up is a suitable approach to develop an upscale industrial process.

### Influence of the genetic modifications on strain productivity

Here we examined the impact of different metabolic engineering strategies and their combinations on α-santalene productivity and yield. The control strain was minimally engineered to produce α-santalene functionally expressing a codon optimized santalene synthase (SanSyn) from *Clausena lansium* and a truncated version of 3-hydroxyl-3-methyl-glutaryl-coenzyme A reductase (HMGR). SanSyn belongs to the class I group of sesquiterpene cyclase. These enzymes catalyze a complex intramolecular cyclization of FPP with very different product specificity and the reaction mechanism often involves several partial reactions [41]. SanSyn has a high specificity for α-santalene as its main product with only minor amounts of *trans*-α-bergamotene formed [12]. Many studies have reported examples of heterologous production of isoprenoids simply expressing the plant synthase in the desired microbial host. However, the yields obtained are often extremely low [15,19,20,42-45]. Similar to our previous study we decided to construct a reference α-santalene producing strain (SCIGS 29) combining the synthase expression with the expression of the deregulated form of Hmg1 (tHmg1) [12]. The use of *tHMG1* represents an excellent example of bypassing one of the regulatory mechanisms controlling the MVA pathway flux and has been successfully applied in a number of microbial isoprenoid production processes [14,19-21,43]. The yield obtained in this control strain was comparable with our previously reported values obtained during a fed-batch process [12] and demonstrates the feasibility and robustness in applying our novel double-phase continuous cultivation. In order to improve the production of the target compound it is necessary to overcome the regulatory mechanisms that have evolved to prevent flux imbalances. In this work, we modulated some of the well-recognized key points that tightly regulate the carbon flux to sesquiterpenes in *S. cerevisiae*. A slight reduction in yield and unchanged productivity observed in the control strain at a higher dilution rate suggests a limitation of the plant synthase in efficiently draining the FPP precursor from the MVA pathway, consistent with the previous hypothesis that at low FPP concentration SanSyn competes with the other cellular FPP consuming reactions [12]. A general strategy extensively applied in sesquiterpene bioprocess development [14,15,20,35] consists in down-regulating SQS to increase the intracellular FPP pool. Replacement of the native P_*ERG9*_ promoter with the glucose-sensing P_*HXT1*_ promoter was recently successfully employed to divert the carbon flux to sesquiterpene products instead of sterols [12]. Applying the same *ERG9* modification in this study together with deletion of *LPP1* greatly increased the sesquiterpene productivity and yield under chemostat conditions compared to the control strain. The obtained productivity level appears to increase with the dilution rate employed pointing to a direct relation between the specific growth rate and the overall flux through the MVA pathway and indicating that the efficiency of the *ERG9* modification in the enhanced FPP availability was supported at different specific growth rates. A similar growth dependent relation has been reported for the cellular content of ergosterol [46], which is also derived from FPP.

In the *lpp1*Δ and *lpp1*Δ/*dpp1*Δ mutants known to exhibit lower FPP phosphatase activity [16,47], the excess of FPP was redistributed between α-santalene and FOH in a consistent ratio when different dilution rates were applied. These results suggest the hypothesis that once a threshold level of intracellular flux toward FPP is reached the thermodynamically favorable endogenous dephosphorylation starts and competes with the catalytic capacity of santalene synthase leading to FOH accumulation. On the other hand, the unchanged α-santalene yield coupled with higher productivity achieved at higher dilution rates suggests that the santalene synthase was not fully saturated at low dilution rates and there was excess activity to cope with high FPP flux. This points out that the FOH formation is not only a direct consequence of limited santalene synthase activity but that other cellular mechanisms are likely to be involved. Reduction but not complete inhibition of FOH formation in the *lpp1*Δ *dpp1*Δ double deletion strain compared to the single *lpp1*Δ deletion strain was consistent with our previous report [12], and confirmed that the *DPP1* encoded lipid phosphate phosphatase has a role in FPP dephosphorylation and together with Lpp1 is involved in the conversion of FPP into FOH. However, these are clearly not the only mechanisms responsible for this conversion as we still observed some FOH production in the double deletion strain, but it is uncertain whether this is the result of the activity of additional phosphatases or caused by non-enzymatic hydrolysis.

Stoichiometry of the pathway reaction for α-santalene formation from glucose reveals a net consumption of 0.4 mol of NADPH and net production of 1.2 mol of NADH per Cmol of α-santalene formed. This fact renders the sequiterpene production pathway a target for cofactor engineering to improve its productivity. Improving the NADPH availability by modifying the ammonium assimilation pathway has proven to be an effective strategy to increase sesquiterpene production [14]. Interestingly, when the previously employed deletion of *GDH1* to manipulate the cell redox metabolism was introduced a reduction in α-santalene productivity without FOH accumulation was obtained. This modification also strongly affected the strain physiology (see below). Therefore, it is likely that the limitation in ammonium assimilation imposed by the *GDH1* deletion reduces the flux through the MVA pathway below the level necessary to trigger FOH formation and conversion of FPP into α-santalene was sufficient to avoid intracellular FPP accumulation.

Combining the simultaneous overexpression of the NAD-dependent glutamate dehydrogenase and prenyl transferase encoded, respectively, by *GDH2* and *ERG20* positively affected sesquiterpene production. Overexpression of *GDH2* is known to restore the ammonium assimilation and consequently alter the NADH:NADPH equilibrium favoring the NADPH availability at the expense of NADH produced [14, 30].

The consensus binding motif for the sterol biosynthesis activating transcription factor Upc2 has been found in most of the promoters of the ergosterol pathway genes [35]. Moreover, it was shown that some genes of the MVA pathway including *ERG8*, *ERG12*, *ERG13*, *ERG20* and *HMG1* contain sequences similar to the consensus binding sequence [32,48]. Expression of *upc2-1* together with an additional copy of *tHMG1* contributed to increase the carbon flux through the MVA pathway and had a beneficial effect on the total sesquiterpene production. The fraction of FOH produced was almost double in this strain and largely contributed to the observed increase of total sesquiterpenes indicating that when the flux toward sesquiterpene is altered through the introduction of genetic modifications the FPP branch point displayed an unexpected flexibility in product distribution.

The optimal solution was obtained through combining all the modifications resulting in the highest sesquiterpene yield (strains SCIGS24 and SCIGS25). Compared to our previous study [12] the engineering strategy employed here led to a 1.8-fold increase in α-santalene final yield (Cmmol α-santalene/Cmmol glucose). These results highlight the importance of combining different engineering strategies to achieve the goal of generating an efficient platform strain for sesquiterpene production. It is noteworthy that comparable sequiterpene productivity was achieved in the strains not fully engineered simply by increasing the operational dilution rate (strains SCIGS29 and SCIGS30) whereas the fully engineered strains were washed out when the same conditions were imposed. Further studies are necessary to elucidate the factors leading to the inability of these mutants to sustain growth at higher dilution rates.

### Influence of genetic modifications on strain physiology

In this study, the effect of controlling the diversion of carbon flow from sterol synthesis towards sequiterpene production by modifying the *ERG9* promoter has been investigated during aerobic chemostat glucose limited cultivation conditions*.* The *lpp1*Δ and *lpp1*Δ/*dpp1*Δ mutants carrying the P_*HXT1*_*ERG9* construct clearly showed an increase in the residual glucose concentration slightly above the critical concentration that triggers aerobic fermentation, which was reported to lie between 0.5 and 0.8 mM [49,50] and results in a typical Crabtree response. It is possible that regulating the Erg9 activity using the P_*HXT1*_ glucose sensitive promoter under strictly glucose limited conditions resulted in its almost complete down-regulation and in an increased biosynthetic demand of the essential compound ergosterol. Ergosterol is the main sterol present in the plasma membranes where it has several essential functions [51]. Yeast is dependent on oxygen for sterol and fatty acid formation. Under strictly anaerobic conditions this compound has to be provided in the media. Reducing its provision results in a decrease of biomass formation and an increase in ethanol formation [52]. Activity of P_*HXT1*_ has been shown to be induced at an extracellular glucose concentration of 5.6 mM [53] suggesting that the observed increase in the residual glucose concentration in the cultures was necessary to restore a minimal P_*HXT1*_ activity in order to maintain the ergosterol level necessary to sustain cell growth. The response to the limitation in the essential compound ergosterol could be the reason leading to the observed decrease in biomass yield and increase of the fermentative metabolism. A similar phenomenon in fact was observed in auxotrophic yeast strains in uracil-limited chemostat culture [54]. The observed overflow metabolism toward ethanol and acetate formation increases the carbon flux through the PDH bypass possibly resulting in an increase in the cytosolic acetyl-CoA availability that was subsequently more efficiently channeled towards the MVA pathway in the engineered strains enhancing the final sesquiterpene production.

Strain SCIGS31 exhibited a particular physiology and needs to be discussed separately. Deletion of *GDH1* is known to impair the ammonium assimilation resulting in a lower specific biomass formation rate on different carbon sources (glucose/galactose) and under different growth conditions (batch/chemostat and aerobic/anaerobic) [29,30], which was confirmed in this study. When deletion of *GDH1* was introduced, ethanol formation as well as glucose accumulation occurred, resulting in a situation similar to cultivation limited in essential nutrients [54]. Most likely, the combination of the limitation in ammonium assimilation as result of the *GDH1* deletion together with the possible ergosterol limitation due to the *ERG9* down regulation produced the observed respiro-fermentative metabolism.

## Conclusions

Microbial production of sesquiterpenes is an active research area; advances in pathway engineering and fermentation technologies have a significant impact in accomplishing the aim to develop an economically viable biobased industrial process. In this study, engineering different pathways simultaneously resulted in a robust *S. cerevisiae* production host capable of efficiently producing α-santalene. The engineered strains were evaluated in an optimized double-phase continuous fermentation method leading to a high yield of α-santalene and resulting in a robust production process that could possibly be used for commercial applications. Levels of products observed open up to the possibility to explore new engineering option for increasing the sesquiterpene productivity. The presented systematic metabolic engineering approach represents a gateway toward the creation of yeast platform that can be applied to the production of an array of sesquiterpene products.

## Methods

### Plasmid construction

An overview of the plasmids constructed in this study is reported in Table 2, the detailed maps of the plasmids are contained in Additional file 1. The gene coding for α-santalene synthase (*SanSyn*_*opt*_) was codon optimized for expression in *S. cerevisiae* and synthesized by DNA 2.0 (Menlo Park, CA, USA) (Additional file 2), cut with *Not*I*/Pac*I and ligated into *Not*I*/Pac*I restricted vector pICK01 containing *tHMG1*[12] resulting in plasmid pISP15 (Figure 1).

**Table 2:** Plasmids used in this study

**Plasmid name**	**Plasmid description**	**Reference**
pSP-GM2	*URA3*-based expression plasmid carrying a bidirectional P_*TEF1*_-P_*PGK1*_ promoter	[55]
pICK01	P_*TEF1*_-*SanSyn*, P_*PGK1*_-*tHMG1*	[12]
pISP15	P_*TEF1*_-*SanSyn*_*opt*_, P_*PGK1*_-*tHMG1*	this study
pIGS01	P_*TEF1*_-*ERG20*	this study
pIGS02	P_*TEF1*_-*ERG20*, AD1	this study
PIGS03	P_*TEF1*_-*ERG20*, P_*PGK1*_-*GDH2*, AD1	this study
pIGS04	P_*TEF1*_-*ERG20*, P_*PGK1*_-*GDH2*, AD1, AD2	this study
pIGS05	P_*TEF1*_-*ERG20*, P_*PGK1*_-*GDH2*, AD1, AD2, *KlURA3*	this study
pIGS06	P_*TEF1*_-*ERG20* P_*PGK1*_-*GDH2*, AD1, 5´*KlURA3*	this study
pIGS07	P_*TEF1*_-*tHMG1*	this study
pIGS08	P_*TEF1*_-*tHMG1*, P_*PGK1*_-*upc2-1*	this study
pIGS09	P_*TEF1*_-*tHMG1*, P_*PGK1*_-*upc2-1*, AD3	this study
pIGS10	P_*TEF1*_-*tHMG1*, P_*PGK1*_-*upc2-1*, AD3, 3´*KlURA3*	this study

To simultaneously integrate multiple genes into the yeast genome a series of plasmids containing the genes, constitutive strong promoters, terminators, marker gene sequences and the required region for genomic integration were constructed. All endogenous *S. cerevisiae* genes were PCR amplified using genomic DNA of strain CEN.PK113-5D as template. Primers used for amplification are provided in Additional file 3. All PCRs were performed using high fidelity Phusion™ DNA polymerase (Finnzymes, Vantaa, Finland). The *ERG20* gene [GenBank: NM_001181600] was amplified using primer pair 1/2, subsequently digested with *Bam*HI*/Nhe*I and ligated into the vector pSP-GM2 [55] restricted with the respective enzymes downstream of the *TEF1* promoter resulting in plasmid pIGS01. A 711 bp upstream flanking region (AD1) selected for genomic integration was amplified using primer pair 3/4, cut with *Mre*I*/Kpn*2I and ligated into vector pIGS01 restricted with the respective enzymes resulting in plasmid pIGS02. Plasmid pIGS03 was obtained by cloning gene *GDH2* [GenBank: NM_001180275] amplified with primer pair 5/6 into pIGS02 downstream of the *PGK1* promoter using *Pac*I*/Not*I restriction sites. A downstream flanking region of 653 bp (AD2) was amplified with primers 7/8, digested with *Asc*I/*Avr*II and ligated into pIGS03. The resulting plasmid was named pIGS04. To complete the plasmid for integration the *Kluyveromyces lactis* (*Kl*) *URA3* gene [GenBank: Y00454] was amplified with primers 9 and 10 using plasmid pWJ1042 [56] as template, cut with *Fse*I and ligated into pIGS04 after restriction with the respective enzyme. The resulting plasmid was designated pIGS05, digested with *Mre*I/*Asc*I and the resulting fragment used for integration into the yeast genome as described below. The 5´ region of the *Kl URA3* gene was amplified with primers 11 and 12, cut with *Avr*II/*Asc*I and cloned into pIGS03 restricted with the respective enzymes resulting in plasmid pIGS06. Amplification of the catalytic domain of the HMG-CoA reductase gene (*tHMG1*) [GenBank: NM_001182434] was performed using primer pair 13/14, the resulting fragment cleaved with *Nhe*I*/Bam*HI and cloned downstream of the *TEF1* promoter into *Nhe*I*/Bam*HI restricted pSP-GM2 resulting in pIGS07. A mutant allele *upc2-1* of the *UPC2* gene [GenBank: NC_001180521] was created by use of primer pair 15/16. To introduce the pleiotropic mutation G888D, the corresponding codon GGT was mutated to GAT generating the amino acid substitution. Subsequently, the PCR amplified *upc2-1* was cloned downstream of the *PGK1* promoter into pIGS07 using *Not*I*/Pac*I resulting in plasmid pIGS08. An 829 bp downstream flanking region (AD3) selected for genomic integration was amplified using primer pair 17/18 cut with *Mre*I*/Kpn*2I and ligated into vector pIGS08 restricted with the respective enzymes resulting in plasmid pIGS09. The 3´ region of *Kl URA3* (overlapping with the 5´region described above) was amplified with primers 19 and 20, cut with *Avr*II*/Asc*I and cloned into pIGS09 restricted with the respective enzymes resulting in plasmid pIGS10. All plasmids were verified by sequencing (Sigma-Aldrich, St. Luis, MO). Subsequently, plasmids pIGS06 and pIGS10 were restricted with *Mre*I*/Asc*I, the cassettes isolated from the vector backbone and used for yeast transformation (see below).

### Yeast strain construction

All *S. cerevisiae* strains constructed in this work have a CEN.PK background with auxotrophy for uracil [57] and are listed in Table 3.

**Table 3:** **List of*S. cerevisiae*strains used in this study**

**Strain**	**Genotype**	**Plasmid**	**Reference**
CEN.PK113-5D	*MAT***a ***MAL2-8*^*c *^*SUC2 ura3-52*	none	P. Kötter, University of Frankfurt, Germany
SCIGS28	*MAT***a ***MAL2-8*^*c *^*SUC2 ura3-52*	pISP15	this study
SCICK01	*MAT***a ***MAL2-8*^*c *^*SUC2 ura3-52 lpp1*Δ::*loxP* P_*ERG9*_Δ::*loxP*-P_*HXT1*_	none	[12]
SCIGS29	*MAT***a ***MAL2-8*^*c *^*SUC2 ura3-52 lpp1*Δ::*loxP* P_*ERG9*_Δ::*loxP*-P_*HXT1*_	pISP15	this study
SCICK16	*MAT***a ***MAL2-8*^*c *^*SUC2 ura3-52 lpp1*Δ::*loxP dpp1*Δ::*loxP* P_*ERG9*_Δ::*loxP*-P_*HXT1*_	none	[12]
SCIGS30	*MAT***a ***MAL2-8*^*c *^*SUC2 ura3-52 lpp1*Δ::*loxP dpp1*Δ::*loxP* P_*ERG9*_Δ::*loxP*-P_*HXT1*_	pISP15	this study
SCIGS03	*MAT***a ***MAL2-8*^*c *^*SUC2 ura3-52 lpp1*Δ::*loxP dpp1*Δ::*loxP* P_*ERG9*_Δ::*loxP*-P_*HXT1*_*gdh1*Δ::*loxP*	none	this study
SCIGS31	*MAT***a ***MAL2-8*^*c *^*SUC2 ura3-52 lpp1*Δ::*loxP dpp1*Δ::*loxP* P_*ERG9*_Δ::*loxP*-P_*HXT1*_*gdh1*Δ::*loxP*	pISP15	this study
SCIGS06	*MAT***a ***MAL2-8*^*c *^*SUC2 ura3-52 lpp1*Δ::*loxP dpp1*Δ::*loxP* P_*ERG9*_Δ::*loxP*-P_*HXT1*_*gdh1*Δ::*loxP* P_*TEF1*_-*ERG20* P_*PGK1*_-*GDH2 KlURA3*	none	this study
SCIGS22	*MAT***a ***MAL2-8*^*c *^*SUC2 ura3-52 lpp1*Δ::*loxP dpp1*Δ::*loxP* P_*ERG9*_Δ::*loxP*-P_*HXT1*_*gdh1*Δ::*loxP* P_*TEF1*_-*ERG20* P_*PGK1*_-*GDH2*	none	this study
SCIGS24	*MAT***a ***MAL2-8*^*c *^*SUC2 ura3-52 lpp1*Δ::*loxP dpp1*Δ::*loxP* P_*ERG9*_Δ::*loxP*-P_*HXT1*_*gdh1*Δ::*loxP* P_*TEF1*_-*ERG20* P_*PGK1*_-*GDH2*	pISP15	this study
SCIGS23	*MAT***a ***MAL2-8*^*c *^*SUC2 ura3-52 lpp1*Δ::*loxP dpp1*Δ::*loxP* P_*ERG9*_Δ::*loxP*-P_*HXT1*_*gdh1*Δ::*loxP* P_*TEF1*_-*ERG20* P_*PGK1*_-*GDH2* P_*TEF1*_-*tHMG1* P_*PGK1*_-*upc2-1*	none	this study
SCIGS12	*MAT***a ***MAL2-8*^*c *^*SUC2 ura3-52 lpp1*Δ::*loxP dpp1*Δ::*loxP* P_*ERG9*_Δ::*loxP*-P_*HXT1*_*gdh1*Δ::*loxP* P_*TEF1*_-*ERG20* P_*PGK1*_-*GDH2* P_*TEF1*_-*tHMG1* P_*PGK1*_-*upc2-1 KlURA3*	none	this study
SCIGS25	*MAT***a ***MAL2-8*^*c *^*SUC2 ura3-52 lpp1*Δ::*loxP dpp1*Δ::*loxP* P_*ERG9*_Δ::*loxP*-P_*HXT1*_*gdh1*Δ::*loxP* P_*TEF1*_-*ERG20* P_*PGK1*_-*GDH2* P_*TEF1*_-*tHMG1* P_*PGK1*_-*upc2-1*	pISP15	this study

Strain SCIGS03 carrying a *GDH1* [GenBank: NC_001183795] deletion was created from strain SCICK16 using a bipartite gene-targeting technique [55]. Upstream and downstream region of *GDH1* were amplified by PCR from CEN. PK113-5D genomic DNA using primer pairs 23/24 and 25/26. The *loxPkanMXloxP* cassette was amplified from plasmid pUG6 [58] as two overlapping fragments using primer pairs 29/30 (5´ part) and 31/32 (3´ part). By fusion PCR, the upstream region of *GDH1* was combined with the 5´ part of the kanMX cassette and the 3´ part of the *kanMX* cassette with the downstream region of *GDH1* and the resulting fragments used to transform SCICK16. Transformation was performed using the standard lithium acetate procedure [59] and transformants were selected using YPD plates containing 200 mg/l G418 (Formedium, Hunstanton, UK). Correct integration of the *kanMX* cassette into the *GDH1* locus was tested by PCR using primers 27/28. The *kanMX* marker was subsequently excised by transient transformation with plasmid pSH47 containing the Cre recombinase encoding gene [58] leading to formation of strain SCIGS03. Strain SCIGS06 carrying a genomic integration of genes *ERG20* and *GDH2* under control of the *TEF1* and *PGK1* promoter, respectively, was obtained by transforming strain SCIGS03 with the *Mre*I*/Asc*I fragment isolated from plasmid pIGS05. Correct integration into the YORWΔ22 locus on chromosome XV [60] was verified by PCR using primer pairs 33/1 and 5/34. Strain SCIGS12 carrying a genomic integration of genes *ERG20*, *GDH2*, *tHMG1* and *upc2-1*, was constructed by co-transforming strain SCIGS03 with the *Mre*I*/Asc*I fragments isolated from plasmids pIGS06 and pIGS10. Correct integration into the YORWΔ22 locus was verified by PCR using primer pairs 33/1, 5/35, 19/15, 35/15, 16/36 and 13/34.

*Kl URA3* was replaced in strains SCIGS06 and SCIGS12 with the *kanMX* marker. The *loxPkanMXloxP* cassette was independently amplified from plasmid pUG6 using primer pair 37/38 for integration in strain SCIGS06 and 39/40 for integration in SCIGS12 containing 71–74 bp primer tails complementary to the target integration sites. Both strains were transformed with the respective PCR-amplified fragment. Transformants were selected on YPD plates containing 200 mg/l G418. *Kl URA3* replacement was initially tested by replica plating on synthetic complete (SC) medium without uracil and YPD/G418 medium. The *kanMX* marker was subsequently removed [58] leading to strains SCIGS22 and SCIGS23.

Strains SCIGS28, SCIGS29, SCIGS30, SCIGS31, SCIGS24 and SCIGS25 were obtained transforming, respectively, strains CEN.PK113-5D, SCICK01, SCICK16, SCIGS03, SCIGS22 and SCIGS23 with the high copy number plasmid pISP15 (Table 3) containing the *URA3* gene and the genes *SanSyn*_*opt*_ and *tHMG1* under control of the strong constitutive promoters *TEF1* and *PGK1*, respectively (Table 2).

### Strain maintenance

Long term storage of yeast suspensions containing 25% (vol/vol) sterile glycerol was performed in cryovials at −80°C [61]. Working stocks were maintained on YPD agar plates containing 10 g/l yeast extract, 20 g/l casein peptone, 20 g/l glucose and 20 g/l agar. Plasmid carrying strains were maintained on synthetic dextrose medium agar plates lacking uracil containing 6.9 g/l yeast nitrogen base without amino acids (Formedium), 0.77 g/l complete supplement mixture without uracil (Formedium) 20 g/l dextrose and 20 g/l agar.

### Media and growth conditions

A mineral salts medium was used for batch cultivations as previously described [62] and had the following composition (per liter): (NH_4_)_2_SO_4_, 5 g; KH_2_PO_4_, 3 g; MgSO_4_·7H_2_O, 0.50 g; Antifoam 289 (A204, Sigma–Aldrich), 0.05 ml; trace metals, 1 ml and vitamins, 1 ml. The trace metal solution consisted of the following (per liter): EDTA (sodium salt), 15.0 g; ZnSO_4_·7H_2_O, 0.45 g; MnCl_2_·2H_2_O, 1 g; CoCl_2_·6H_2_O, 0.3 g; CuSO_4_·5H_2_O, 0.3 g; Na_2_MoO_4_·2H_2_O, 0.4 g; CaCl_2_·2H_2_O, 0.45 g; FeSO_4_·7H_2_O, 0.3 g; H_3_BO_3_, 0.1 g and KI, 0.1 g. The pH of the trace metal solution was adjusted to 4.0 with 2 M NaOH prior to heat sterilization. The vitamin solution contained (per liter): biotin, 0.05 g; p-amino benzoic acid, 0.2 g; nicotinic acid, 1 g; Ca-pantothenate, 1 g; pyridoxine-HCl, 1 g; thiamine-HCl, 1 g and myo-inositol, 25 g. The pH of the vitamin solution was adjusted to 6.5 with 2 M NaOH. The vitamin solution was filter sterilized and stored at 4°C. This medium was supplemented with 20 g/l glucose. The feed composition used for continuous cultivation had the same composition as described above, but the glucose concentration was 10 g/l. The medium used for shake flask cultivation has the same composition as described above, but the (NH_4_)_2_SO_4_ concentration was increased to 7.5 g/l, and the KH_2_PO_4_ to 14.4 g/l; the glucose concentration was 20 g/l; the pH was adjusted to 6.5 prior autoclaving.

### Inoculum preparation and pre-culture

A single colony from an SC-ura agar plate was selected to inoculate a 500 ml shake flask containing 100 ml mineral salts medium. The seed culture was grown at 30°C in an orbital shaker at 100 rpm to late-exponential phase and used to inoculate the fermenter to a final dry weight of 1 mg/l. All cultivations were performed in triplicate.

### Chemostat operation

Aerobic, carbon limited chemostat cultivations were performed in 1.0 l stirrer pro vessels (DasGip, Jülich, Germany) with a working volume of 0.3 l. The temperature was monitored using a platinum RTD temperature sensor and kept at 30°C using a BioBlock integrated heating and cooling thermo well. Agitation was maintained at 600 rpm using an overhead drive stirrer with one Rushton impeller. The air flow rate was kept at 1 vvm by a mass flow controller (DasGip). The pH was maintained constant at 5.0 by automatic addition of 2 M KOH. The fermenters were integrated in a DasGip monitor and control system used to control all fermentation parameters, temperature, agitation, pH, and gas flow. Dissolved oxygen was monitored using an autoclavable polarographic oxygen electrode (Mettler Toledo, Columbus, OH) and maintained above 30% saturation via regulating stirrer speed and gas flow rate. Exhaust gas was cooled, dried and the gas composition was analyzed for real time continuous determination of oxygen and carbon dioxide concentration by a DasGip fed batch pro® gas analysis system with off gas analyzer GA4 based on zirconium dioxide and two-beam infrared sensor. The integrated mass flow sensor allowed on-line monitoring and calculation of oxygen transfer rate (OTR), carbon dioxide transfer rate (CTR) and respiratory quotient (RQ). The chemostat bioreactor was initiated as batch culture with 10 g/l glucose. Only after the residual ethanol produced was completely consumed the feed was started and the fermentation run in a continuous mode. Fermenters were operated at dilution rate 0.05 or 0.1 h^-1^. A two-phase product partition chemostat was performed by co-feeding medium containing 10 g/l glucose and the organic phase (Figure 2). To obtain a dilution rate of 0.1 h^-1^, the inlet medium was fed at 27 ml/h and the organic phase at 3 ml/h. To obtain a dilution rate of 0.05 h^-1^, medium was fed at 13.5 ml/h and the organic phase at 1.5 ml/h resulting in a constant inlet feed ratio of medium: organic phase of 9:1 (vol/vol). Dodecane (Sigma-Aldrich, St. Luis, MO) was used as organic phase and filter sterilized prior addition. The culture working volume of 0.3 l (0.27 l of medium + 0.03 l of dodecane) was kept constant by automatic withdrawal of broth based on an electric level sensor measurement. The set-up allowed maintaining the correct medium/organic phase ratio inside the fermented throughout the fermentation time. The correct ratio of 9:1 vol/vol between the two phases was constantly monitored and differed by less than 2% in samples taken directly from the culture and from the effluent line. Steady state was reached after at least 5 residence times, defined by constant values of CTR, OTR and biomass concentration (less than 5% deviation).

### Cell mass determination

Cell growth during fermentation was monitored off-line by measuring optical density and dry cell weight and on-line with an optical density transmitter OD4 sensor (DasGip) integrated in the fermenter system. The optical density at 600 nm was determined using a Genesis20 spectrophotometer (Thermo Scientific, Madison, WI, USA). The cell dry weight was measured by filtering known culture volumes through pre-dried and pre-weighed 0.45-μm-pore size nitrocellulose filters (Sartorious Stedim Biotech GmbH, Göttingen, Germany). The filters with the biomass were washed with water, dried for 15 min in a microwave oven at 150 W, and weighed again. The correlation factor between off-line and on-line parameters was determined.

### Metabolite analysis

Samples for analysis of extracellular metabolite concentrations were withdrawn from two-phase steady state chemostat cultures and centrifuged for 5 min at 5000 g. The organic layer was discarded and the cultivation broth was filtered through 0.45-μm-pore size nylon filters (VWR international, Radnor, PA, USA) and stored at −20°C until further analysis. Glucose, glycerol acetate, succinate, and pyruvate were quantified by HPLC (UltiMate® 3000 Nano, Dionex, Bannockburn, IL, USA) with an Aminex HXP-87 H ion-exchange column (Bio-Rad, Hercules, CA) maintained at 65°C and using 5 mM H_2_SO_4_ as mobile phase at a flow rate of 0.6 ml min^-1^. Glucose, glycerol, and ethanol were measured with a refractive index detector (Shodex RI-101, Showa Denko, Tokyo, Japan), and acetate, succinate, and pyruvate were measured with a UV-visible light absorbance detector (UltiMate 3000 Variable Wavelength Detector, Dionex).

### Analysis of sesquiterpenes

Sequiterpene production was determined as described previously [12] with minor modifications. Culture samples were centrifuged 15 min at 5000 g and the organic layer was diluted with an equal volume of dodecane containing a defined amount of α-humulene as internal standard. Samples were diluted in heptane and analyzed by gas chromatography–mass spectrometry (Thermo Scientific) equipped with an SLB-5 ms capillary column (30 m, 0.25 mm i.d., 0.25 μm film thickness; Supelco, Bellefonte, PA, USA). Full mass spectra were generated by scanning the *m/z* range within 40–500 for metabolite identification. Sesquiterpene identification was carried out comparing mass spectra and retention time with authentic standards, concentrations were calculated using a correction factor determined for the internal standard α-humulene relative to α-santalene and *EE*-farnesol.

## Abbreviations

CTR: Carbon dioxide transfer rate; DMAPP: Dimethylallyl diphosphate; ER: Endoplasmic reticulum; FOH: Farnesol; FPP: Farnesyl diphosphate; FPPS: Farnesyl diphosphate synthase; GPP: Geranyl diphosphate; HMGR: 3-hydroxy-3-methyl-glutaryl-CoA reductase; IPP: Isopentenyl diphosphate; ISPR: *In situ* product removal; MVA: Mevalonate; OTR: Oxygen transfer rate; PDH: Pyruvate dehydrogenase; RQ: Respiratory quotient; SanSyn: Santalene synthase; SQS: Squalene synthase.

## Competing interests

GS, SP, VS and JN declare they have no competing interests. MS and LD are employees of Firmenich SA.

## Authors' contributions

JN and GS participated in the design of the study. JN and VS supervised the project. GS performed the experimental work. SP assisted in the molecular biology experiments. MD and LD assisted in the GC/MS analysis of sesquiterpens. GS analyzed the data and wrote the manuscript. All the authors discussed the results, edited and approved the final manuscript.

## Supplementary Material

Additional file 1 **Maps of plasmids constructed in this study.** (A) pISP15, (B) pIGS01, (C) pIGS02, (D) pIGS03, (E) pIGS04, (F) pIGS05, (G) pIGS06, (H) pIGS07, (I) pIGS08, (L) pIGS09, (M) pIGS10. Click here for file

Additional file 2 Codon optimized santalene synthase nucleotide sequence. Click here for file

Additional file 3 **Primers used in this study.** Restriction sites are indicated in bold face, overlapping nucleotides are underlined, and the modified codon is indicated in italic. Click here for file
