# Supplementary material for: Combined metabolic engineering of precursor and co-factor supply to increase α-santalene production by Saccharomyces cerevisiae
Source: Microb Cell Fact. 2012 Aug 31;11:117. doi: 10.1186/1475-2859-11-117 (PMC3527295; doi:10.1186/1475-2859-11-117)
Supplement: Additional file 1 — Maps of plasmids constructed in this study. (A) pISP15, (B) pIGS01, (C) pIGS02, (D) pIGS03, (E) pIGS04, (F) pIGS05, (G) pIGS06, (H) pIGS07, (I) pIGS08, (L) pIGS09, (M) pIGS10. [file 1475-2859-11-117-S1.pdf]

Additional file 1 – Maps of plasmid constructed in this study

(A) pISP15, **(B)** pIGS01, **(C)** pIGS02, **(D)** pIGS03, **(E)** pIGS04, **(F)** pIGS05, **(G)** pIGS06, **(H)** pIGS07, **(I)** pIGS08, **(L)** pIGS09, **(M)** pIGS10.

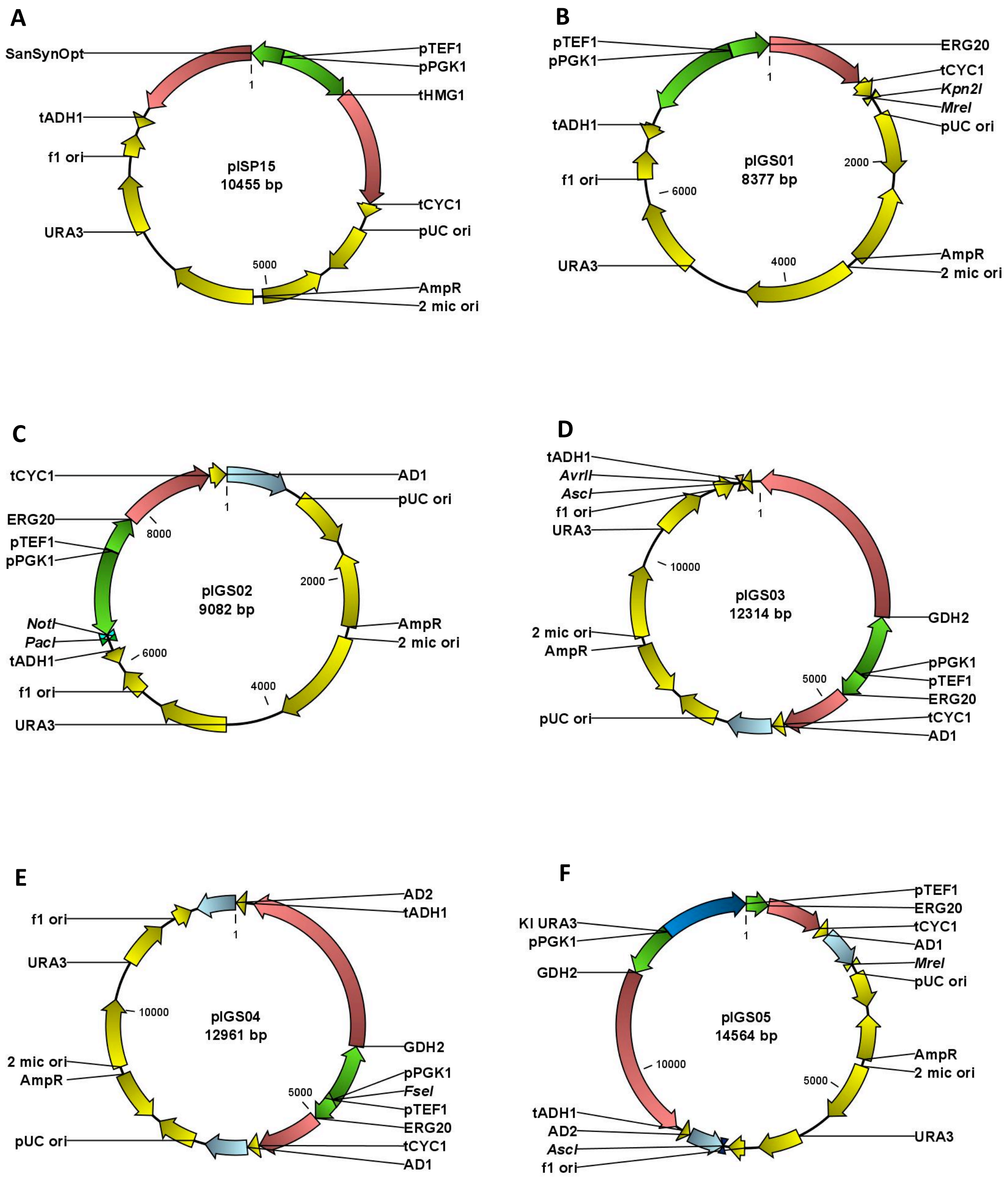

G

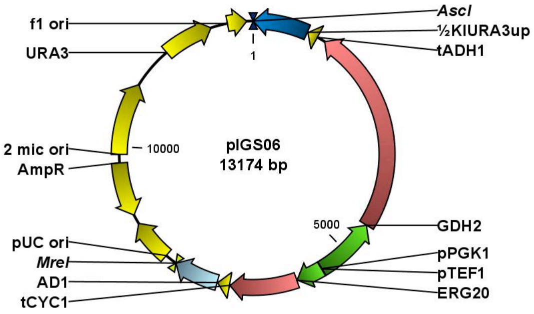
f1 ori
URA3
2 mic ori
AmpR
pUC ori
MreI
AD1
tCYC1
AscI
½KlURA3up
tADH1
GDH2
pPGK1
pTEF1
ERG20
1
10000
5000
pIGS06
13174 bp

H

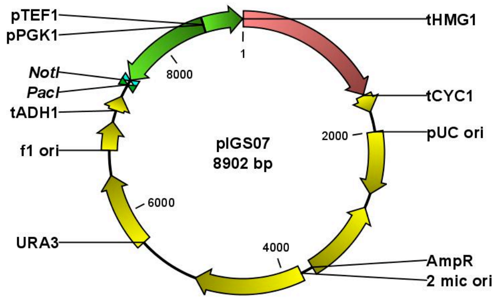
pTEF1
pPGK1
NotI
PacI
tADH1
f1 ori
URA3
tHMG1
tCYC1
pUC ori
AmpR
2 mic ori
1
8000
2000
6000
4000
pIGS07
8902 bp

I

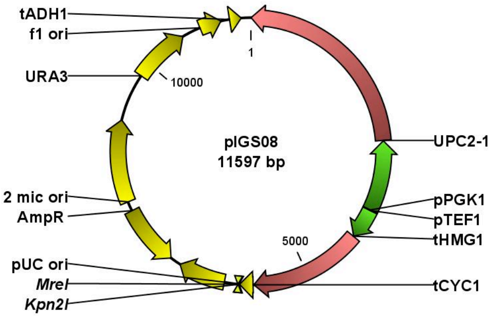
tADH1
f1 ori
URA3
2 mic ori
AmpR
pUC ori
MreI
Kpn2I
UPC2-1
pPGK1
pTEF1
tHMG1
tCYC1
1
10000
5000
pIGS08
11597 bp

L

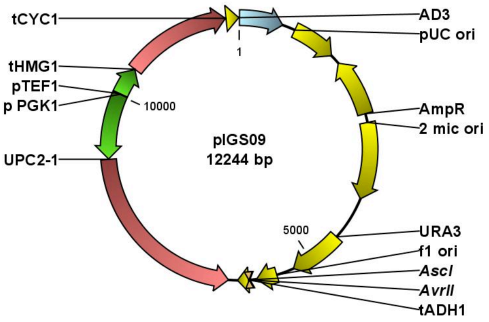
tCYC1
tHMG1
pTEF1
p PGK1
UPC2-1
AD3
pUC ori
AmpR
2 mic ori
URA3
f1 ori
AscI
AvrII
tADH1
1
10000
5000
pIGS09
12244 bp

M

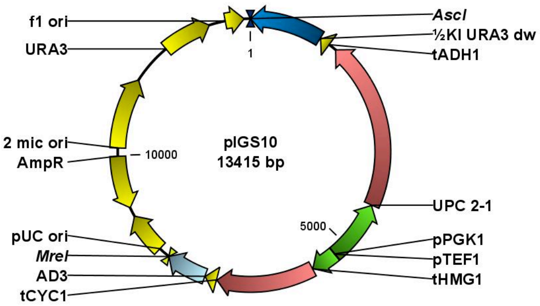
f1 ori
URA3
2 mic ori
AmpR
pUC ori
MreI
AD3
tCYC1
AscI
½Kl URA3 dw
tADH1
UPC 2-1
pPGK1
pTEF1
tHMG1
1
10000
5000
pIGS10
13415 bp
