# Supplementary material for: Combined metabolic engineering of precursor and co-factor supply to increase α-santalene production by Saccharomyces cerevisiae
Source: Microb Cell Fact. 2012 Aug 31;11:117. doi: 10.1186/1475-2859-11-117 (PMC3527295; doi:10.1186/1475-2859-11-117)
Supplement: Additional file 2 — Codon optimized santalene synthase nucleotide sequence. [file 1475-2859-11-117-S2.pdf]

Additional file 2 –Codon optimized santalene synthase nucleotide sequence

ATGTCTACTCAACAAGTCTCATCCGAGAACATAGTTAGAAATGCTGCGAACTTTCATCCT
AACATATGGGGTAACCATTTTCTGACTTGTCCTTCTCAAACAATTGACTCTTGGACCCAAC
AGCACCACAAGGAACTTAAGGAAGAGGTGCGAAAGATGATGGTTTCTGATGCTAACAAA
CCAGCGCAAAGATTGCGTTTGATTGATACCGTTCAAAGGCTGGGTGTTGCCTACCATTTTG
AAAAGGAAATTGATGATGCTTTAGAAAAGATTGGTCATGATCCTTTCGATGACAAAGATG
ATTTGTACATCGTTTCACTATGTTTCAGACTCCTTCGTCAACACGGTATCAAGATCTCCTG
TGATGTGTTTGAAAAGTTCAAAGATGACGATGGAAAGTTCAAGGCCAGCCTGATGAATG
ACGTCCAAGGTATGCTATCATTGTACGAAGCAGCTCATTTGGCAATCCATGGGGAAGATA
TCTTGGATGAAGCAATAGTGTTTACTACTACTCACTTAAAGTCCACAGTTTCTAATTCACC
AGTCAATAGTACATTTGCTGAACAGATAAGACACTCTTTGAGAGTTCCATTGAGGAAAGC
TGTCCCAAGATTGGAGTCTAGGTATTTCCTTGATATCTACTCCAGAGATGACTTACACGAC
AAAACACTCTTAAACTTTGCTAAACTAGATTTCAACATCCTACAGGCTATGCACCAAAAG
GAAGCTAGTGAAATGACCAGATGGTGGAGAGATTTTGATTTCCTCAAAAAGCTTCCTTAC
ATTAGAGATCGAGTGGTTGAGTTGTACTTCTGGATTTTGGTTGGTGTAAGCTATCAACCAA
AGTTCAGTACGGGACGTATCTTCCTTAGTAAGATTATCTGCCTCGAAACATTGGTAGACG
ATACCTTCGATGCCTATGGAACTTTTGATGAACTAACAATCTTCACTGAGGCTGTAACTAG
ATGGGATATTGGCCATAGAGATGCCTTGCCAGAATACATGAAGTTTATCTTCAAAACACT
CATTGATGTATACTCTGAGGCCGAACAAGAGCTGGCCAAGGAAGGTAGATCTTACTCAAT
TCAATATGCTATAAGATCATTCCAGGAGCTAGTAATGAAATACTTTTGTGAAGCAAAGTG
GTTGAATAAGGGCTACGTGCCATCTCTAGATGACTACAAATCCGTCTCATTGCGTTCAATC
GGTTTCCTGCCAATCGCAGTGGCCTCTTTTGTTTTCATGGGGGACATAGCGACAAAGGAA
GTTTTTGAATGGGAAATGAACAATCCTAAGATAATCATTGCAGCAGAGACGATATTCAGA
TTCCTCGATGATATTGCGGGACATAAGTTTGAGCAGAAGAGAGAACATTCCCCTTCCGCA
ATTGAATGCTACAAAAACCAACATGGTGTCTCAGAGGAGGAGGCTGTCAAAGCTTTATCT
TTAGAAGTAGCAAACTCTTGGAAAGACATAAACGAGGAATTACTTCTGAATCCAATGGCC
ATTCCTTTACCTTTACTTCAAGTTATCTTAGACTTATCTAGAAGCGCTGACTTCATGTACG
GCAATGCACAAGATAGACTTACACACTCAACTATGATGAAAGATCAAGTCGATTTGGTAC
TAAAAGATCCAGTTAAGTTAGATGATTAG
